# New Appliances and Things Medical

**Published:** 1896-02-22

**Authors:** 


### NEW APPLIANCES AND THINGS MEDICAL.

[We shall be glad to receive, at our Office, 428, Strand, London, W.C., from the manufacturers, specimens of all new preparations and appliances, which may be brought out from time to time.]

### PARMENA BISCUITS.

(Macfarlane, Lang, and Co., Glasgow, London, and Bristol.)

The manufacturers have sent us specimens of these biscuits, which are flavoured with Parmesan cheese and are an excellent sample. Having carefully tested them we can confidently recommend them for general use; they are a very valuable addition to the table, being specially suitable for lunch, and the manufacturers regularly supply them to the households of the Queen and Prince of Wales. We know of no biscuit calculated to prove more popular, or helpful, or appetising.

### EXTRA DRY CHAMPAGNE.

(Arnold, Perrett, and Co., Limited, 7a, Lower Belgrave Street, S.W.)

This champagne, which is specially shipped for the above celebrated firm, is an Epernay wine made by Alix et Cie. It is known as the "Carte Noire," and, being of peculiarly dry quality, is highly serviceable for those of gouty or rheumatic diathesis, who find benefit in the stimulating and nutritive properties of a good champagne, which have no saccharine or unconverted sugary elements. For convalescents or for general invalid purposes the Carte Noir is a perfectly safe champagne, and we highly recommend it to the notice of hospitals and nursing institutions as well as to private individuals.

